# The posterior cerebellum and inconsistent trait implications when learning the sequence of actions

**DOI:** 10.1093/scan/nsab037

**Published:** 2021-03-26

**Authors:** Min Pu, Qianying Ma, Elien Heleven, Naem Patemoshela Haihambo, Frank Van Overwalle

**Affiliations:** Faculty of Psychology and Center for Neuroscience, Vrije Universiteit Brussel, Pleinlaan 2, Brussels 1050, Brussels, Belgium; Faculty of Psychology and Center for Neuroscience, Vrije Universiteit Brussel, Pleinlaan 2, Brussels 1050, Brussels, Belgium; Faculty of Psychology and Center for Neuroscience, Vrije Universiteit Brussel, Pleinlaan 2, Brussels 1050, Brussels, Belgium; Faculty of Psychology and Center for Neuroscience, Vrije Universiteit Brussel, Pleinlaan 2, Brussels 1050, Brussels, Belgium; Faculty of Psychology and Center for Neuroscience, Vrije Universiteit Brussel, Pleinlaan 2, Brussels 1050, Brussels, Belgium

**Keywords:** posterior cerebellum, internal models, sequence learning, inconsistent trait updating

## Abstract

It has been proposed that the cerebellum contributes to social cognition. Based on the view that cerebellar internal models create predictions on motions and actions, we hypothesize that the posterior cerebellum supports identifying temporal sequences of persons’ actions as well as detecting inconsistent actions that violate the implied trait. Participants were required to memorize the temporal order of a set of sentences that implied a personality trait. Importantly, the sentence sets were designed in such a way that the first half of each set involved actions that were consistent with the same trait, while the other half was either consistent or inconsistent with that trait. As expected, we found robust posterior cerebellar activation when memorizing the order of the actions, irrespective of trait consistency, but more crucially also for actions implying an inconsistent trait in comparison to consistent trait actions. We also found that the medial prefrontal cortex and posterior cerebellum were associated with confidence level in retrieving the sequences. This study supports the hypothesis that the posterior cerebellum identifies and predicts the low-level temporal order of actions and demonstrates for the first time that this area is also involved in the high-level prediction of trait implications of those actions.

## Introduction

Successful social interaction requires making inferences about the minds of other people, termed social mentalizing. Social mentalizing includes the desires, traits and beliefs of others and helps people to predict and evaluate the behaviors of others. Traits are enduring personality characteristics (Mitchell *et al.*, 2006), and we often make trait inferences about what kind of a person someone is, for instance, is she or he honest or unreliable? However, social interaction is a dynamic process, where we continually update and revise initial impressions in light of new behavioral information, such as social action that is inconsistent with prior behavior (Ma *et al.*, 2012; Mende-Siedlecki *et al.*, 2013b).

Although prior neuroimaging research on impression updating has mainly focused on the role of the cerebral cortex (Schurz *et al.*, 2014), the contribution of the cerebellum remained largely unexplored. Traditionally, it has been assumed that the cerebellum is a coordinator of sensorimotor function (Wiestler *et al.*, 2011). However, a more advanced function evolved, which allowed the cerebellum to construct internal models of pure mental states without the involvement of sensorimotor responses (Ito, 2008; Pisotta and Molinari, 2014). Although only a few past functional magnetic resonance imaging (fMRI) studies on social mentalizing specifically targeted the cerebellum, Van Overwalle *et al.* (2014) found consistent cerebellar activation in tasks involving high-level abstract social inferences in a large-scale meta-analysis including over 350 fMRI studies.

These studies suggest that the cerebellum crucially contributes to social cognition. However, the specific function of the cerebellum in cognition and mentalizing remains unaddressed. One influential theory is the ‘sequence detection’ hypothesis (Leggio and Molinari, 2015). According to this hypothesis, the cerebellum detects repetitive patterns of temporally or spatially structured events and makes predictions via internal models constructed during learning and experience, irrespective of whether the events involve motor planning or higher-order mental processes (e.g. social cognition).

Is the cerebellum also involved in action sequences that require understanding the mental state of others, including beliefs and traits, as recently proposed by Van Overwalle and colleagues (Van Overwalle *et al.*, 2019a)? With respect to others’ beliefs, a recent pilot study by Van Overwalle *et al.* (2019a) demonstrated that cerebellar patients showed deficits in reconstructing the correct sequence of randomly ordered cartoon-like pictures that required mentalizing about the belief of others compared to routine social scripts and non-social mechanical sequences. An fMRI study exploring the role of the cerebellum in the same task revealed stronger activation in the posterior cerebellum (Crus 1 and 2) during the identification of action sequences involving others’ belief compared to non-social mechanical sequences (Heleven *et al.*, 2019). Furthermore, a recent study using dynamic causal modeling revealed a significant pattern of bidirectional connectivity linking the posterior cerebellum to traditional mentalizing areas recruited during belief inferences (e.g. temporoparietal junction, TPJ) (Van Overwalle *et al.*, 2020), suggesting that the cerebellum builds internal predictive models of action sequences in close synchrony with cortical mentalizing areas.

Although these previous studies demonstrated that the cerebellum is recruited when social actions carry an inherently logical and necessary sequence (Heleven *et al.*, 2019; Van Overwalle *et al.*, 2019a), a recent fMRI study further indicated that the cerebellum is also activated when memorizing social actions in an arbitrary temporal sequence (Pu *et al.*, 2020). The study revealed significantly stronger posterior cerebellar Crus activation in social trait sentences in comparison to several control sentences involving non-social sequencing and non-sequencing events. However, all actions in that study implied the same personality trait of the person. Therefore, it remains unclear whether cerebellar activation was due to the processing of specific trait implications or of the general social context regardless of any traits. This raises questions on the specific role of the cerebellum with respect to inferences about others’ traits.

To address this question, the present study investigates action sequences that either confirm or violate the implied trait. Indeed, as noted earlier, social interaction requires flexible updating of impressions in view of novel behaviors that violate earlier impressions. Therefore, we predict that the cerebellar function of generating internal models of social actions may contribute not only to identifying social actions in the temporal order as perceived (i.e. low level), but also to detecting violations of social expectations, such as their trait implications (i.e. high level). Consequently, we expect greater involvement of the cerebellum when behavioral actions contradict rather than confirm traits implied by previous behaviors (Van Overwalle *et al.*, 2019b).

To investigate this prediction, the present study used a memory paradigm slightly modified from the previous study by Pu *et al.* (2020). As in that study, participants were required to memorize the given temporal order of a series of social actions that implied a personality trait of a person. The actions implied the same trait (consistent condition) as in the previous study, and additional actions implied the opposite trait (inconsistent condition). Participants were then asked to recall the correct order of social actions learned earlier. In addition, we included a non-social non-sequencing control condition in which sentences implied a feature of an object rather than a trait of a person. We hypothesize that the posterior cerebellar Crus will be recruited while learning social action sequences, regardless of consistent or inconsistent trait implications, as opposed to a non-social non-sequencing control condition. More importantly, we hypothesize that the posterior cerebellar Crus, as an error-detecting system also at a higher social level, will be recruited more when participants read the actions involving inconsistent as opposed to consistent traits.

Additionally, previous studies suggested that metacognition recruits a domain-general network involving the medial and lateral prefrontal cortices, as well as local brain areas related to the specific task like meta-memory and meta-decision (McCurdy *et al.*, 2013; Vaccaro and Fleming, 2018; Pu *et al.*, 2020). We further investigate the role of the cerebellum during metacognitive confidence in retrieving the social sequences.

## Method

### Participants

Twenty-six (18 females; age mean ± SD, 23 ± 2 years old) healthy right-handed, native Dutch-speaking volunteers were recruited to participate in this fMRI study. All participants had normal or corrected-to-normal vision and reported no neurological or psychiatric disorders. Informed consent was obtained with the approval of the Medical Ethics Committee at the Hospital of University of Ghent, where the study was conducted. Participants were paid 20 euros in exchange for their participation and transportation costs.

### Stimulus materials

We presented sets of six sentences each. In the consistent condition, trait-implying sentence sets described a fictitious protagonist engaging in a series of behaviors from which a trait could be strongly inferred, i.e. ‘gives a hug to the new colleague’ implies the personality trait of ‘friendly’. Half of the sentence sets involved positive behaviors (positive-to-positive updating condition) and the other half involved negative behaviors (negative-to-negative updating condition). In the inconsistent condition, trait-implying sentence sets comprised social actions with either four or five behaviors implying a consistent trait and remaining two or one behaviors implying an inconsistent opposite trait. In half of the sets, the consistent trait was positive that turned into a negative inconsistent trait (positive-to-negative updating condition), while in the other half the reverse order was presented (negative-to-positive updating condition). To create a strong trait prediction, the inconsistent trait sentences were always presented after the third sentence. Half of the inconsistent sets included one inconsistent trait sentence at a random position in sentences 4–6 and the other half included two inconsistent trait sentences also at random positions in sentences 4–6 with the provision that they were adjacent to each other. The consistent and inconsistent conditions together compose the social condition. As a comparison, in the control condition, non-social sentence sets described a common feature of an object, i.e. ‘goes up higher into the sky’ implies the feature ‘floating’ of an object, which in this case is a balloon.

We used the same non-social object-implying sentences and social consistent trait-implying sentences used in previous studies (Ma *et al.*, 2014a; Pu *et al.*, 2020). The inconsistent trait sentences, which imply the opposite trait, were adapted from earlier trait-implying research (Ma *et al.*, 2014a) and were sometimes newly created by the authors of this study. In a pilot study, to test the applicability of the inconsistent trait sentences, participants (*n* = 25) were asked to rate how applicable the opposite trait was with respect to the behavioral sentences, using a 7-point scale (1* =*‘not applicable at all’, 4* =*‘neutral’ and 7* =*‘very applicable’). Inconsistent trait sentences were selected when the applicability rating was >5.5. All selected sentences contained 5–10 words, with most sentences containing seven words.

### Procedure

**Fig. 1.**
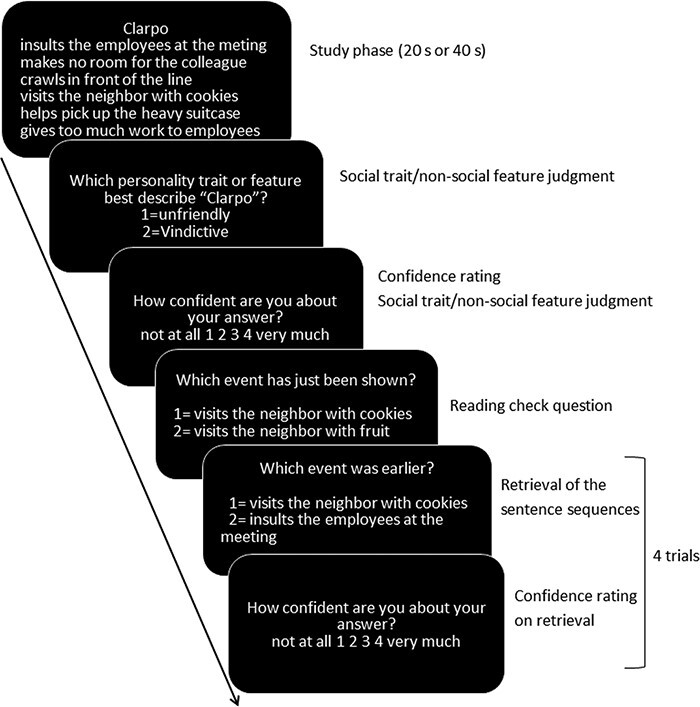
Experimental procedure. Participants were instructed to learn the given temporal order of a set of six sentences involving a single person or object and had to infer from these six sentences a common trait of the person or feature of the object. An example of the inconsistent sequence condition is shown here (i.e. the fourth and fifth sentences imply an opposite trait ‘friendly’ of the prior consistent trait ‘unfriendly’). All questions were preceded by a blank screen with a fixation cross in the center, which was jittered randomly between 0 s and 2 s, and ratings had to be answered within 5 s.

We used the same procedure as in Pu *et al.* (2020). In brief, participants were instructed to learn a given temporal order of a set of six sentences involving a single person or object and had to infer from these sentences a common trait of that person or feature of the object. Participants were presented with 16 social consistent trait sets (consistent condition), 16 social inconsistent trait sets (inconsistent condition) and 8 non-social object-feature sets (control condition). The sentence sets were presented in a random order within the social (consistent and inconsistent) condition and the control condition. In each set, the order of the sentences had to be learned in 20 s for half of them and in 40 s for the other half (randomly determined). The two different durations were intended to have different levels of difficulty to generate varying levels of recognition accuracy and confidence. Before the actual experiment, participants performed several practice trials. For each sentence set of the social conditions, the same procedure was followed (see Figure 1):

Specifically, during the study phase, participants were instructed to learn and memorize the correct temporal order of a set of sentences. First, the name of the protagonist would appear on the top of the screen and six sentences were shown on screen one-by-one (1.1 s after the previous sentence). Sentences were then presented together for a total duration of 20 s or 40 s. A red notice appeared on the top of the screen to indicate that 10 s remained before the task ended. To optimize the estimation of the event-related fMRI response for inconsistent sentences, a mean 500 ms jitter (randomly ranging between 0 ms and 1000 ms) was presented between the first and second half of each sentence set regardless of conditions.

Afterwards, participants were asked ‘Which personality trait describes the person best?’ Two options were given in a random order: one option was the correct trait and the other was a distractor with the same valence. Participants were then asked to rate how confident they were about their trait judgment using a 4-point rating scale (1 = ‘not at all’ and 4 = ‘very much’). Next, to verify whether participants had read and understood the sentences, they were asked to respond to a check question where they had to indicate ‘Which of the two sentences was shown before?’ and where one sentence came from the sentences set just presented, and the other was a slightly reworded version.

Finally, during the retrieval phase, participants were instructed to recall the correct order of the sentences (see Supplementary Materials).

At the start of the experiment, to provide a general baseline, a control condition involving non-social objects was introduced. Participants were required to read sets of object-feature sentences without memorizing their order. Although these sets had to be read in 20 or 40 s, participants were allowed to end the trial earlier once they were done reading all the sentences, since simply reading the sentences typically took less time. All other aspects of the procedure were identical to the social condition, with the exception of not having a retrieval task and subsequent confidence rating.

All questions and ratings had to be answered within 5 s and were preceded by a blank screen with a fixation cross in the center, which were jittered randomly between 0 ms and 2000 ms (mean = 1000 ms). All responses were given on a response box used with the (non-dominant) left hand. Overall, the participants missed 6.6% and 5.5% of the retrieval trials in the consistent and inconsistent conditions, respectively. They missed 0.5% and 0.3% of the confidence ratings in the memory task in the consistent and inconsistent conditions, respectively. These missed trials were excluded from the behavioral and fMRI analysis.

### Imaging procedure and preprocessing

Images were collected with a Siemens MAGNETOM Prisma fit scanner system (Siemens Medical Systems, Erlangen, Germany) using a 64-channel radiofrequency head coil. Stimuli were projected onto a screen at the end of the magnet bore that participants viewed by way of a mirror mounted on the head coil. Stimulus presentation was controlled by E-Prime 2.0 (https://www.pstnet.com/eprime; Psychology Software Tools) running under Windows XP. Participants were placed head first and supine in the scanner bore and were instructed not to move their heads to avoid motion artifacts. Foam cushions were placed within the head coil to minimize head movements. First, a high-resolution anatomical images were acquired using a T1-weighted 3D MPRAGE sequence (Repetition Time [TR] = 2250 ms, Echo Time [TE] = 4.18 ms, Inversion Time [TI] = 900 ms, Field of View [FOV] = 256 mm, flip angle = 9º, voxel size = 1 × 1 × 1 mm). Second, a fieldmap was calculated to correct for inhomogeneities in the magnetic field (Cusack and Papadakis, 2002). Third, whole-brain functional images were collected in a single run using a T2*-weighted gradient echo sequence, sensitive to Blood Oxygenation Level Dependent (BOLD) contrast (TR = 1000 ms, TE = 31.0 ms, FOV = 210 mm, flip angle = 52º, slice thickness= 2.5 mm, distance factor = 0%, voxel size = 2.5 × 2.5 × 2.5 mm, 56 axial slices, acceleration factor GeneRalized Autocalibrating Partial Parallel Acquisition (GRAPPA) = 4).

SPM12 (Wellcome Department of Cognitive Neurology, London, UK) was used to process and analyze the fMRI data. To remove sources of noise and artifact, data were preprocessed. Inhomogeneities in the magnetic field were corrected using the fieldmap (Cusack and Papadakis, 2002). Functional data were corrected for differences in acquisition time between slices for each whole-brain volume, realigned to correct for head movement, and co-registered with each participant’s anatomical data. Then, the functional data were transformed into a standard anatomical space (2 mm isotropic voxels) based on the ICBM152 brain template (Montreal Neurological Institute, MNI). Normalized data were then spatially smoothed (6 mm full-width at half-maximum) using a Gaussian kernel. Finally, using the Artifact Detection Tool (http://web.mit.edu/swg/art/art.pdf; http://www.nitrc.org/projects/artifact_detect), the preprocessed data were examined for excessive motion artifacts and for correlations between motion and experimental design, and between global mean signal and experimental design. Outliers were identified in the temporal differences series by assessing between-scan differences (*Z*-threshold: 3.0 mm, scan-to-scan movement threshold: 0.5 mm, rotation threshold: 0.02 radians). These outliers were omitted from the analysis by including a single regressor for each outlier. A default high-pass filter of 128s was used and serial correlations were accounted for by the default auto-regressive AR(1) model.

### Statistical analysis of neuroimaging data

#### Whole-brain analysis of memorizing.

The general linear model (GLM) of SPM12 (Wellcome Department of Cognitive Neurology, London, UK) was used to conduct the analyses of the fMRI data. For the GLM at the first (single participant) level, the event-related design was modeled with one regressor for each condition. During the study phase, onsets were specified at the presentation of all sentences-at-once of the sentence set. After the study phase, onsets were specified at the presentation of each question (trait, trait confidence, sequence retrieval and confidence of sequence retrieval) for each of the two social conditions (consistent and inconsistent conditions). As mentioned earlier, missed trials were not modeled. Each regression was convolved with a canonical hemodynamic response function of which the duration was set to 0 s for all questions and confidence ratings after the study phase. During the study phase, duration was determined in the same manner as the study by Pu *et al.* (2020): During the reading baseline, event duration for reading all sentences was set to 4 s (on average the shortest reading time to understand the sentences). Sentence sets with reading time shorter than 4 s were excluded from analysis, and the mean rejection rate of sentence sets was 2.9% (SD = 7.3%). During the social conditions, encoding the sentence order would take about 10 s, and consequently the event duration was set to 10 s for memorizing the order of the sentences.

At the second (group) level, for all study phases and all questions, we conducted a one-way within-subject ANOVA and defined all possible *t*-contrasts of interest comparing baseline control, consistent and inconsistent conditions during the study phase, and comparing trait question, confidence trait question, sequence retrieval and confidence on retrieval question of the consistent and inconsistent conditions.

#### Whole-brain analysis of trait updating.

We then examined brain activity associated with inconsistent trait updating in more detail in the consistent and inconsistent conditions. Recall that each sentence set was split up in a first half of consistent sentences and a second half of mixed consistent and inconsistent sentences. To analyze inconsistency detection in more detail, for the inconsistent condition, we compared the last sentence of the consistent half (third consistent sentence) with the first inconsistent sentence of the second half (first inconsistent sentence). Sentence order was an attempt to have sentences from each halves close to each other, to find a sudden inconsistency effect. To examine trait suppression, for the consistent condition, we compared the first consistent sentence of the first half of the sentence set with first consistence sentence of the second half (fourth consistent sentence). Sentence order attempted to have sentences from each halves more apart from each other, to find a prolonged repetition effect (see also Pu *et al.*, 2020). This resulted in six regressors of interest involving two sentences in the positive-to-negative updating condition, the negative-to-positive updating condition and the consistent condition.

At the second (group) level, the relevant inconsistent > consistent sentences contrasts were specified to investigate the role of the cerebellum and were analyzed as indicated above. In addition, to ensure that trait inferences were made, we conducted the same repetition suppression analysis as Pu *et al.* (2020) for the consistent condition, by conducting a first consistent sentence (prime) > fourth consistent sentence (target) contrast (Ma *et al.*, 2014a; Heleven and Van Overwalle, 2016). Additionally, we analyzed the brain activity of confidence on trait attribution, sequence retrieval and confidence on retrieval phase, reported in Supplementary Materials.

For all whole-brain analyses, the contrasts from the single-subject first-level analyses were entered into a second-level random-effects analysis. Significant brain activations maps were defined at *P *< 0.001, uncorrected with a minimum cluster extent of 10 voxels, and we restricted the analysis to clusters with a Family Wise Error (FWE) corrected cluster-wise threshold *P* < 0.05.

#### Regions of interest analysis.

To explore some of our hypotheses in more detail, we additionally analyzed several regions of interest (ROIs). For trait updating, ROIs for the cerebellum were taken from earlier studies on social cognition, Crus 2 (±24 −76 −40) (Van Overwalle *et al.*, 2019c, 2020). ROIs for social mentalizing were derived from prior meta-analyses on social cognition (Van Overwalle, 2009; Van Overwalle and Baetens, 2009) and involved the following areas and center coordinates: TPJ, ±50 −55 25; precuneus, 0 −60 40; medial prefrontal cortex (mPFC), 0 50 20; and dorsal medial prefrontal cortex (dmPFC), 0 50 35. To investigate the parametric relationship between metacognition and cerebellar activation, we used cerebellar ROIs from an earlier study (Crus 2: 12 −76 −36; Pu *et al.*, 2020). A sphere of 15 mm radius around the centers was used to perform a small volume correction using the same cluster-defining threshold as the whole-brain analysis, with *P *< 0.001, uncorrected with a minimum of 10 voxels. Significant ROIs were identified using a threshold of *P* < 0.05, FWE corrected at the cluster level.

## Results

### Behavioral results

Of most importance, the accuracy of retrieval of sentence order was significantly higher in the inconsistent (mean ± SD: 77% ± 9%) than in the consistent condition (mean ± SD: 73% ± 10%), *t*(25) = −2.4, *P* = 0.024. Additional analyses on trait questions and metacognition are reported in Supplementary Materials.

### Neuroimaging results

#### Memorizing the temporal sequences of social actions.

**Fig. 2.**
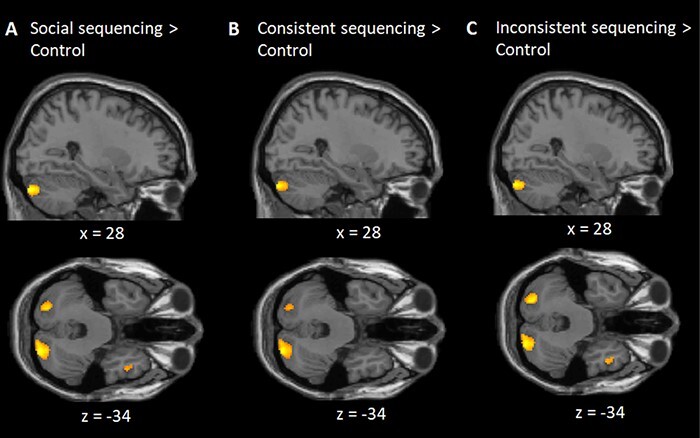
Study (memorizing) phase of social action sequences: Sagittal and transverse views of the contrasts at an uncorrected threshold of *P* < 0.001. The results show that the posterior cerebellum (Crus 1 and 2) was significantly activated in the contrast of (A) social (consistent and inconsistent) sequencing, (B) consistent sequencing, (C) inconsistent sequencing, each in contrast with non-social non-sequencing control. All contrasts, *P* < 0.01, FWE corrected.

First, to identify the brain areas involved in a general sequencing learning effect, we compared the social conditions (i.e. across consistent and inconsistent conditions) against the control condition. The results from a whole-brain analysis revealed significant posterior cerebellar activation (Crus 2) in this contrast. Additional brain activations were found in the bilateral middle occipital gyrus, precuneus, superior and middle temporal gyrus, mPFC and bilateral TPJ (Table 1, Figure 2A). Splitting the data by condition, in the consistent condition, the posterior cerebellum (Crus 2) was significantly activated in the same contrast against the control condition, together with activation in the same cortical areas (but without ventral medial prefrontal cortex, vmPFC), and additionally in the insula and anterior cingulate cortex (Table 1, Figure 2B). In the inconsistent condition, the same contrast revealed significant brain activations in the posterior cerebellum (Crus 1 and 2) as well as the same (sub)cortical areas (Table 1, Figure 2C). Next, we directly compared the consistent against the inconsistent conditions and found significant brain activation in the precuneus, but no significant brain activation in the reverse contrast.

**Table 1.** Whole-brain analysis of action sequencing during the study phase

	MNI coordinate				
Contrasts and Anatomical Label	*x*	*y*	*z*	Voxels	Max. *t*
Sequencing > Non-sequencing control					
R Cerebellum (Crus 2)	28	−86	−34	299	5.51***
R Cerebellum (Crus 2)	16	−90	−32		4.47***
L Middle occipital gyrus	−40	−70	8	805	7.48***
R Middle occipital gyrus	42	−66	4	508	5.70***
L Precuneus	−4	−56	28	931	7.51***
R Superior temporal gyrus	50	−34	20	210	4.72**
L Middle temporal gyrus	−56	−16	−16	250	4.99**
R Middle orbital gyrus	0	52	−12	214	5.08**
L Superior medial gyrus	−6	56	36	568	5.46***
ROI: R TPJ	52	−64	22	199	4.59***
ROI: L TPJ	−48	−58	22	368	5.19***
ROI: mPFC	−2	52	34	54	4.15*
Consistent sequencing > Non-sequencing control					
R Cerebellum (Crus 2)	28	−86	−34	294	5.49**
R Cerebellum (Crus 2)	16	−90	−32		4.59**
L Middle occipital gyrus	−40	−70	8	842	7.85***
R Middle temporal gyrus	42	−66	4	548	5.59***
L Precuneus	−4	−56	28	924	7.11***
R Superior temporal gyrus	50	−34	20	420	5.11***
L Middle temporal gyrus	−58	−16	−16	167	4.88*
R Insula	38	−2	18	147	4.88*
L Anterior cingulate cortex	0	52	−12	228	4.92**
L Superior medial gyrus (mPFC)	−6	56	36	319	5.00***
Inconsistent sequencing > Non-sequencing control					
R Cerebellum (Crus 2)	28	−86	−34	255	5.15**
R Cerebellum (Crus 2)	16	−90	−32		4.06**
L Cerebellum (Crus 1)	−26	−78	−32	138	4.98*
L Middle occipital gyrus	−40	−70	8	663	6.64***
R Middle temporal gyrus	42	−66	4	381	5.43***
L Precuneus	−4	−56	28	818	7.39***
L Middle temporal gyrus	−56	−16	−16	301	4.79***
R Middle temporal gyrus	60	−6	−20	168	4.79*
L Middle orbital gyrus (vmPFC)	0	54	−12	178	5.00*
L Superior medial gyrus (mPFC)	−6	56	38	588	5.58***
Consistent sequencing > Inconsistent sequencing					
L Precuneus	−14	−56	58	301	4.24***
Inconsistent sequencing > Consistent sequencing					
**—**					

Notes: Coordinates refer to the MNI stereotaxic space. Whole-brain analysis thresholded at voxel-wise uncorrected *P* < 0.001 with cluster-wise FWE corrected *P* < 0.05, with voxel extent ≥ 10. Only the highest peaks of each cluster are shown, except for the cerebellum showing all peaks. L = left, R = right.

* *P* < 0.05

** *P* < 0.01

*** *P* **< 0.001 (all significance levels are cluster-level FWE corrected; for ROIs: cluster-level FWE corrected using a small volume correction using a sphere with 15 mm radius and centered around a priori MNI coordinates: TPJ [±50 −55 10], mPFC [0 50 20]).

#### Inconsistent trait updating.

**Fig. 3.**
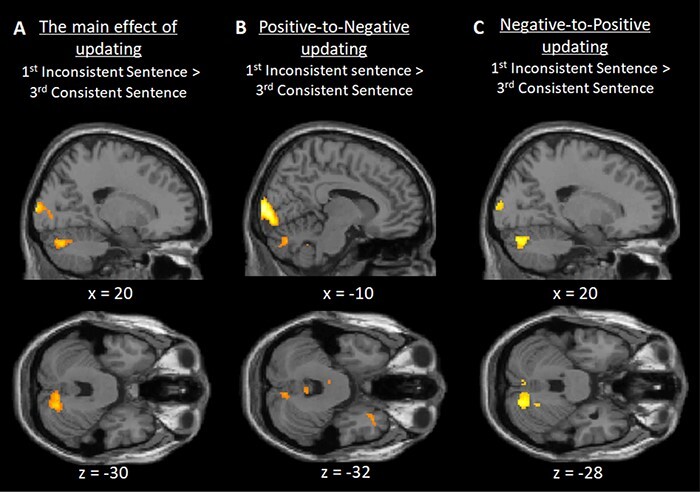
Inconsistent trait updating: Sagittal and transverse views of the contrasts at an uncorrected threshold of *P* < 0.001. The results show that the posterior cerebellum (Crus 1) was significantly activated in the (A) main effect of inconsistent trait updating in the contrast of first inconsistent sentence > third consistent sentence, as well in the separate (B) positive-to-negative and (C) negative-to-positive trait conditions. All contrasts, *P* < 0.05, FWE corrected, except *P* = 0.063 for the positive-to-negative condition.

We then investigated the cerebellar contribution of detecting inconsistent trait-implying actions. For the inconsistent condition, we performed a contrast between the first inconsistent trait sentence in the second half of the sentence sets (first inconsistent sentence) and the third consistent trait sentence in the first half of the sentence sets (third consistent sentence). This analysis demonstrated stronger activation in the posterior cerebellum (Crus 1), inferior frontal gyrus including the lateral PFC, precuneus, TPJ, dmPFC, cerebellum lobule IX, middle frontal gyrus and calcarine gyrus (Table 2, Figure 3A). As expected, a reverse contrast (third consistent sentence > first inconsistent sentence) revealed no significant brain activation in the cerebellum, but revealed activations in the middle occipital gyrus, middle cingulate cortex, and superior and middle temporal gyrus.

**Table 2.** Whole-brain and ROI analysis of trait updating

	MNI coordinate				
Contrasts and Anatomical Label	*x*	*y*	*z*	Voxels	Max. *t*
Inconsistent trait updating					
*Main effect of updating*					
Third consistent sentence > First inconsistent sentence					
L Middle occipital gyrus	−40	−70	8	344	6.37***
R Middle temporal gyrus	46	−66	6	631	6.69***
R Superior temporal gyrus	60	−38	22	457	5.62***
R Middle cingulate cortex	8	−34	42	136	4.75*
First inconsistent sentence > Third consistent sentence					
R Cerebellum (Crus 1)	12	−72	−28	438	4.61***
R Cerebellum (Crus 1)	20	−76	−30		4.43***
R Cerebellum (VI)	24	−66	−26		4.33***
L Calcarine gyrus	−8	−90	−6	2363	9.77***
R Cerebellum (IX)	6	−52	−36	128	5.59*
L Middle frontal gyrus	−42	10	56	123	4.37*
L Inferior frontal gyrus, including lateral PFC	−48	28	28	129	4.53*
ROI: Precuneus	−2	−58	44	66	4.36**
ROI: dmPFC	−4	42	44	20	3.49*
ROI: TPJ	−48	−60	28	31	3.55*
*Negative-to-positive*					
Third consistent sentence > First inconsistent sentence					
L Middle temporal gyrus	−40	−68	8	151	5.47*
R Middle temporal gyrus	44	−66	2	405	5.29***
First inconsistent sentence > Third consistent sentence					
R Cerebellum (Crus 1)	20	−78	−28	163	4.44*
R Cerebellum (VI)	24	−60	−26		3.97*
L Calcarine gyrus	−8	−90	−6	1267	7.47***
*Positive-to-negative*					
Third consistent sentence > First inconsistent sentence					
L Middle occipital gyrus	−42	−72	8	149	4.84*
R Middle temporal gyrus	46	−66	8	248	5.06***
R Supramarginal gyrus	58	−30	34	217	4.21**
First inconsistent sentence > Third consistent sentence					
R Cuneus	12	−98	6	1942	7.55***
R Middle temporal gyrus	62	−14	−24	219	4.18**
L Middle frontal gyrus	−44	10	54	151	4.56*
ROI: R Cerebellum (Crus 2)	12	−72	−32	13	3.40^+^
Consistent trait suppression					
First consistent sentence (Prime) > Fourth consistent sentence (Target)					
vmPFC	8	46	−4	3047	5.61***
R Cuneus	10	−78	40	2136	6.43***
L Middle occipital gyrus	−42	−70	8	355	5.74***
R Fusiform gyrus	30	−68	−14	1065	5.98***
L Fusiform gyrus	−22	−54	−14	686	5.58***
L Superior parietal lobule	−28	−42	72	233	4.34**
R Postcentral gyrus	24	−40	56	243	4.45**
R Supramarginal gyrus	60	−32	24	2594	6.08***
L Supramarginal gyrus	−62	−30	26	275	4.43***
L Superior temporal gyrus	−42	−18	−2	611	4.79***
R Superior frontal gyrus	20	−6	70	136	4.37*
R Middle frontal gyrus	42	46	14	247	4.17***
R Middle frontal gyrus	26	52	30	269	4.49***
Inconsistent and consistent trait sequences in the second half of the sentence set					
Main effect: First inconsistent sentence of inconsistent condition > First consistent sentence of consistent condition					
R Cuneus	8	−80	36	129	4.87*
R Lingual gyrus	20	−58	−6	152	4.63*
ROI: L cerebellum (Crus 1)	−22	−68	−32	45	4.19*
Negative-to-positive: First inconsistent sentence of inconsistent condition > First consistent sentence of consistent condition					
ROI: L cerebellum (Crus 1)	−22	−68	−32	41	4.03*
Positive-to-negative: First inconsistent sentence of inconsistent condition > First consistent sentence of consistent condition					
ROI: L cerebellum (Crus 1)	−24	−70	−32	10	3.41^+^

Notes: Coordinates refer to the MNI stereotaxic space. Whole-brain analysis thresholded at voxel-wise uncorrected *P* < 0.001 with cluster-wise FWE corrected *P* < 0.05, with voxel extent ≥ 10. Only the highest peaks of each cluster are shown. L = left, R = right.

+ *P* < 0.10

* *P* < 0.05

** *P* < 0.01

*** *P* **< 0.001 [all significance levels are cluster-level FWE corrected; for ROI using a small volume correction using a sphere with 15 mm radius and centered around a priori MNI coordinates: Cerebellum Crus 2 (±24 −76 −40), TPJ (±50 −55 10), precuneus (0–60 40) and dmPFC (0 50 35)].

We then compared the effect of inconsistent trait updating (first inconsistent sentence > third consistent sentence) separately for positive-to-negative and negative-to-positive conditions. We found significantly stronger posterior cerebellar activation (Crus 1) for negative-to-positive updating (Table 2, Figure 3C), and a trend for positive-to-negative updating (Table 2, Figure 3B). This finding reflects the role of the cerebellum in updating trait impressions based on inconsistent behaviors. Additionally, to control for the potential mitigating effects of sentence position, we compared the sentences in the second half only, that is, the first inconsistent > first consistent sentence of the second half, in the inconsistent and consistent conditions, respectively. These contrasts again showed significant cerebellar activations in Crus 1 in the negative-to-positive condition, and a trend in the positive-to-negative condition (Table 2).

Furthermore, to provide evidence of trait mentalizing during the consistent condition, we performed a trait suppression contrast (first consistent sentence > fourth consistent sentence). The results revealed significant brain activation in the ventral part of mPFC (Table 2). Additional activations from the whole-brain analysis were found in the cuneus, fusiform gyrus, supramarginal gyrus, middle frontal gyrus, middle occipital gyrus, superior parietal lobule, postcentral gyrus, superior temporal gyrus and superior frontal gyrus.

## Discussion

Our study aimed to assess the hypothesis that the posterior cerebellar Crus, by generating internal prediction models, contributes to explicit learning of temporal sequences of actions that imply a person’s trait and supports detection of actions that violate the trait implication (i.e. prediction error). As expected, compared to a non-social non-sequencing control condition, the posterior cerebellar Crus 2 was activated when participants learned and memorized the temporal sequences of trait-implying social actions. More importantly, as hypothesized, there was a significant increase in activation of the posterior cerebellar Crus 1 as soon as the action implied a trait that was inconsistent as opposed to consistent with the trait implied by previous actions.

In agreement with the functional role of the posterior cerebellar Crus in social sequence learning, our results confirm our hypothesis that learning sequences of trait-implying actions triggers significant activation in the posterior cerebellar Crus. This is a core area of the mentalizing network responsible for processing social action sequences linked to mental inferences, such as true or false beliefs held by other persons (Heleven *et al.*, 2019; Van Overwalle *et al.*, 2019b) and connecting the cerebellum with the cerebral cortex during mental inferences (Van Overwalle *et al.*, 2020). This cerebellar activation was observed in comparisons with a non-social non-sequence control condition, regardless of whether the implied trait was consistent or inconsistent with previous trait implications. This finding supports a recent study, which demonstrated robust posterior cerebellar Crus 2 activation for consistent trait-implying sequence learning as opposed to a non-sequence control condition (Pu *et al.*, 2020), and extends it to inconsistent trait inferences. Together, the prior and present studies provide evidence on the inherent role of sequencing for social inferences by the posterior cerebellar Crus, regardless of whether these inferences violate or confirm prior inferences. They further demonstrate that the cerebellar Crus is activated, even when the sequences of social actions are arbitrary and imposed on the participants, not only when the sequences of social actions are inherently logical (Heleven *et al.*, 2019).

More importantly, our results further demonstrate, for the first time, that activation in the posterior cerebellar Crus 1 was systematically enhanced when actions were inconsistent with previous trait implications. Specifically, while the first half of similar trait-implying actions generated trait-specific expectancies of a person, distinct trait actions presented in the second half (in the inconsistent condition) violated this social prediction, leading to stronger cerebellar activation. This cerebellar activation, given trait violations, did not depend on the order of the actions, but most probably reflected the processing of error signals used to update trait predictions on the person (i.e. people expect others to behave consistently with regard to their personality traits). This confirms our hypothesis that the posterior cerebellar Crus encodes social actions, not only at the observed low-level temporal order, but also at the high-level trait implications.

Although prior neuroimaging research revealed cerebellar activation during social cognition (Van Overwalle *et al.*, 2014), little was known about the specific contribution of the cerebellum in these social mentalizing processes, such as trait inconsistency detection and resolution. The present finding of stronger activation after trait inconsistencies in a series of actions is consistent with current theoretical perspectives, which propose that the cerebellum uniformly creates internal models in the sensorimotor and cognitive domain that serve to learn and predict future events and send error signals (Braitenberg *et al.*, 1997; Ito, 2006, 2008; Ramnani *et al.*, 2006; Leggio and Molinari, 2015). Our study provides novel evidence on the role of the cerebellum in detecting violations at another social cognitive level: the traits implied by actions. This suggests that prediction is a critical cerebellar function that operates across various task domains.

One may argue that this study and the prior study by Pu *et al.* (2020) are limited in that the temporal sequence of the actions did not depend on any inherent logical order, unlike previous cerebellar studies where the temporal sequence of actions was an inherent part mandated by the action elements themselves (Leggio *et al.*, 2008; Heleven *et al.*, 2019). Although the temporal orders were imposed on the participants, they had to identify and predict these orders because they had to memorize them, which recruited the cerebellum. This is very similar to the motor domain such as car driving, where an initial temporal order of technical manipulations may appear arbitrary to a novice, but become later an inherent and ‘logical’ part of car driving for an expert.

For cortical activations, this study confirms prior evidence that core social mentalizing areas, such as the TPJ, precuneus and mPFC (Van Overwalle and Baetens, 2009; Schurz *et al.*, 2014), were recruited when learning social action sequences (Pu *et al.*, 2020). Although precuneus activation was observed when participants memorized both consistent and inconsistent action sequences, unlike the other mentalizing regions, stronger activation was observed for consistent than for inconsistent action sequences. This supports the view that the precuneus is linked to scene construction of general situational models, through which participants can tie all actions together (Hassabis and Maguire, 2007; Palombo *et al.*, 2018; Costigan *et al.*, 2019). Apparently, participants appeared to do this more intensely when all actions implied the same trait, which could easily lead to a coherent mental scene representation, and less so when the actions implied opposite traits, most likely because such a coherent mental construction was difficult to achieve.

Furthermore, when participants updated the trait implications of behavioral inconsistencies, apart from cerebellar areas, mentalizing regions (e.g. dmPFC, TPJ and Precuneus; Van Overwalle, 2009) and domain-general conflict monitoring region (e.g. lateral PFC; Carter and Van Veen, 2007; Zaki *et al.*, 2010) were strongly activated (Ma *et al.*, 2012; Mende-Siedlecki and Todorov, 2016). Interestingly, the dmPFC is not only responsible for trait mentalizing, but also for updating inconsistent trait impressions (Ma *et al.*, 2012; Mende-Siedlecki *et al.*, 2013b) and general evaluative inconsistency (Cloutier *et al.*, 2011). In brief, these findings suggest that inconsistent trait implications are not only strongly activated in the cortical network (e.g. mentalizing and conflict monitoring regions), but also engaged in subcortical regions (e.g. cerebellum). However, future studies should further investigate the effective connectivity between the cerebrum and cerebellum during these processes. In addition, our findings confirm previous studies that found decreased ventral mPFC activation in trait repetition suppression (Heleven and Van Overwalle, 2019).

Limitations of this study should be noted. First, the cerebellar contribution to inconsistent trait updating was limited to the negative-to-positive condition and showed only a trend in the positive-to-negative condition. Future research is needed to test the robustness of a cerebellar role in updating violated traits. Second, our study was somewhat limited in that our non-sequential control condition involved non-social objects and their features, and not social actions and their trait implications, like in the main social conditions. The main reason we did not include a social non-sequential condition is that trait-implying actions may potentially activate the cerebellum to some degree even without sequencing information, because traits often do imply some order (e.g. gallantry implies that you let others pass first; aggression implies that you hit first and not in response to others’ harm). Therefore, such a social non-sequential condition would not constitute an entirely valid and strong baseline control (Van Overwalle *et al.*, 2014; Pu *et al.*, 2020).

## Conclusion

Overall, our findings suggest an important role of the posterior cerebellar Crus in learning sequences of social trait-implying actions. Moreover, for the first time, this study provides novel evidence that the posterior cerebellar Crus is also involved in detecting violations in the implied trait of actions, which reflects the cerebellar contribution to higher-level implications in social cognition. Together, our results indicate that the posterior cerebellar Crus encodes internal predictions not only of perceived temporal structures, but also of higher mentalizing implications (i.e. traits; Van Overwalle *et al.*, 2019a).

## Supplementary Material

nsab037_Supp
