# Erratum to: The posterior cerebellum and inconsistent trait implications when learning the sequence of actions

**DOI:** 10.1093/scan/nsab091

**Published:** 2021-07-29

**Authors:** Min Pu, Qianying Ma, Elien Heleven, Naem Patemoshela Haihambo, Frank Van Overwalle

**Affiliations:** Faculty of Psychology and Center for Neuroscience, Vrije Universiteit Brussel, Brussels 1050, Belgium; Faculty of Psychology and Center for Neuroscience, Vrije Universiteit Brussel, Brussels 1050, Belgium; Faculty of Psychology and Center for Neuroscience, Vrije Universiteit Brussel, Brussels 1050, Belgium; Faculty of Psychology and Center for Neuroscience, Vrije Universiteit Brussel, Brussels 1050, Belgium; Faculty of Psychology and Center for Neuroscience, Vrije Universiteit Brussel, Brussels 1050, Belgium

In the originally published version of this manuscript, incorrect supplementary data was included.

The correct supplementary data is here.

The Publisher apologises for these errors.

