# Supplementary material for: The posterior cerebellum and inconsistent trait implications when learning the sequence of actions
Source: Soc Cogn Affect Neurosci. 2021 Mar 26;16(7):696–706. doi: 10.1093/scan/nsab037 (PMC8259289; doi:10.1093/scan/nsab037)
Supplement: nsab037_Supp [file nsab037_supp.zip › scan-20-340-File006.docx]

**Supplementary Materials for**

**The posterior cerebellum and inconsistent trait implications when learning the sequence of actions**

Min Pu, Qianying Ma, Elien Heleven, Naem Patemoshela Haihambo and Frank Van Overwalle

Faculty of Psychology and Center for Neuroscience, Vrije Universiteit Brussel, Belgium

**This supporting material includes information on retrieval, retrieval confidence/meta-cognition and trait attributions:**

1. **Method**
   - Procedure on retrieval and confidence (during the retrieval phase)
   - Neuroimaging and behavioral analyses of retrieval and confidence (during the retrieval phase)
2. **Results**
   - Behavioral results on trait judgment and metacognition
   - Neuroimaging results on trait attribution, retrieval and confidence (during the retrieval phase)
3. **Discussion** on retrieval and metacognitive confidence
4. **Table S1**: neuroimaging results about retrieval and confidence (during the retrieval phase)

**Method**

**Procedure on retrieval and confidence (during the retrieval phase)**

During the retrieval phase, participants were instructed to recall the correct order of the sentences consisting of four trials by answering the question “which of the two sentences were shown earlier during the study phase (1 = *the first sentence*, 2 = *the second sentence*)”, and to rate how confident they were about their retrieval accuracy (*metacognitive* phase), using a 4-point scale (1 = *not at all*, 4 = *very much*). On each trial, two sentences (sentence pairs) were shown in a random order. For the consistent condition, the sentence pairs were selected randomly from the study phase. For the inconsistent condition, half of the sentence pairs were selected randomly from the consistent trait sentences, while the other half contained one or two inconsistent trait sentences.

**Neuroimaging and behavioral analyses**

***Whole-brain analysis of retrieval***

The same whole-brain analysis for retrieval was used as for memorizing (see Method section)

***Whole-brain parametric analysis of retrieval accuracy and confidence***

To detect whether the brain activity covaries with retrieval accuracy or with confidence ratings, another two GLM models were specified to estimate BOLD responses at the first-level parametric analysis. Specifically, retrieval accuracy (correct and incorrect) and confidence rating were included in first-level analyses as a modulator on a trial-by-trial basis (Morales, Lau, & Fleming, 2018). Separate regressors were created for each Social (e.g., Consistent and Inconsistent) condition. In a subsequent analysis, we split up these regressors according to the duration of the presentation of the sentence sets (20 versus 40 seconds) because the previous study by (Pu et al., 2020) demonstrated that duration had a differential effect on the modulation of the posterior cerebellar activation (and also on individual metacognitive sensitivity, see below). We ran two parametric models: During the retrieval phase with accuracy of retrieval as parametric modulator; and during the confidence rating phase with confidence rating as modulator. Single-subject contrast images of the parametric modulator were entered into a second-level random-effects analysis which was similar to the one described above.

To further investigate whether brain activation during sequence retrieval was correlated with individuals’ retrieval accuracy, and whether activation during confidence rating was correlated with individual indices of metacognitive sensitivity (e.g., meta-ratio), each participant’s mean retrieval accuracy was entered as a covariate in a second-level regression analysis, and participant’s meta-ratio index was entered as covariate in a second-level regression analysis in each Consistent and Inconsistent condition (Bègue et al., 2019; Pu et al., 2020).

***Behavioral analyses***

We examined accuracy of the selected trait/feature, retrieval memory (quantified as the % correct responses), and manipulation check questions. We used a paired *t*-test on the accuracy of all questions above with consistency (Consistent vs. Inconsistent) as independent variables. With regard to the metacognitive judgment, we used the meta-d' calculation (Fleming & Lau, 2014; Maniscalco & Lau, 2012) on participants’ confidence ratings, using publicly available scripts (<http://www.columbia.edu/~bsm2105/Type2sdt/>). We also calculated meta-ratio (meta-d'/d') which tests the efficacy of metacognitive sensitivity by qualifying the degree to which confidence ratings discriminate between correct and incorrect trials unconfounded by first-order performance.

**Results**

**Behavioral results**

The average accuracy on the check question in the Social and Control conditions was 84% (SD = 10%) and 79% (SD = 9%), respectively. For trait questions, we found a significantly higher accuracy in the Consistent (mean ± SD: 93% ± 7%) than in the Inconsistent condition (mean ± SD: 87% ± 9%), *t*(25) = 2.58, *p* = 0.016. Likewise, for confidence ratings on trait judgment, we found significantly higher confidence in the Consistent (mean ± SD: 3.48 ± 0.29) than Inconsistent condition (mean ± SD: 3.01 ± 0.35), *t*(25) = 6.94, *p* < 0.001. The accuracy of objects’ feature judgments in the Control condition was 91% (SD = 7%).

For metacognitive judgments on the trait questions, we found no significant difference on the meta-ratio (e.g., meta-d'/d') between Consistent (mean ± SD: 0.96 ± 0.65) and Inconsistent conditions (mean ± SD: 0.94 ± 0.72), *t*(25) = 0.08, *p* = 0.93. Likewise, for metacognitive judgments on sequence retrieval, we found no significant difference on meta-ratio between Consistent (mean ± SD: 1.65 ± 1.86) and Inconsistent conditions (mean ± SD: 1.27 ± 0.56), *t*(25) = 1.2, *p* = 0.24.

**Neuroimaging results**

***Confidence on trait attribution***

Note that for confidence on trait judgments, we had no specific hypotheses. In the regular factorial analysis, no significant brain activation was found. For the first-level parametric analysis, we found a positive correlation between the confidence ratings and the activation in the calcarine gyrus in the Consistent condition, and the striatum (i.e., caudate and putamen) in the Inconsistent condition. No other significant correlations were found.

***Sequence retrieval***

No significant brain activation was found for the general linear model using a regular factorial SPM analysis. We then conducted a first-level parametric analysis between retrieval accuracy and brain activity. We found activity in the calcarine gyrus correlated positively with retrieval accuracy in the Consistent condition. No other correlations were found for other conditions. No significant difference of brain activity between these parametric regressors at the second level (e.g., Consistent > Inconsistent) was found. No significant brain activation was found after a second-level regression analysis on retrieval accuracy.

Next, we split up the Consistent and Inconsistent condition according to the duration of the presentation of the sentence sets (20 versus 40 seconds; **Table S1**). In the factorial analysis, the contrast of the Consistent 40 seconds > Consistent 20 seconds condition revealed significant activation in the cerebellar lobule Ⅷ. The contrast of Inconsistent 20 seconds > Inconsistent 40 seconds condition showed activations in the insula, medial temporal gyrus, postcentral gyrus and superior frontal gyrus. For the first-level parametric analysis, activation in the precuneus correlated positively with retrieval accuracy in the Consistent 20 seconds condition, while activations in the cuneus and posterior-medial frontal cortex correlated with accuracy in the Inconsistent 20 seconds condition. From the second-level regression analyses, we found that activation in the superior parietal lobule correlated positively with retrieval accuracy in the Inconsistent 20 seconds condition.

Together, as hypothesized, no significant associations of sequence retrieval with activation in the posterior cerebellum of interest were found.

***Confidence on sequence retrieval accuracy***

No significant brain activation was found for the general linear model using a regular factorial SPM analysis. In a first-level parametric analysis, for the Consistent condition, we found significant positive correlations between confidence ratings and brain activity in the lingual gyrus and precentral gyrus, while the brain activity in the caudate, hippocampus, precentral gyrus, middle temporal and occipital gyrus, and lingual gyrus correlated positively with confidence ratings in the Inconsistent condition (**Table S1**). Additionally, an Inconsistent > Consistent contrast on these regressors at the second level revealed significant brain activation in the right caudate (**Table S1**), whereas no significant activation was found for the reverse Consistent > Inconsistent contrast. No significant brain activations were found for second-level regression analyses on metacognitive sensitivity (e.g., meta-ratio). Together, no significant associations with the cerebellum were found, even after applying a ROI analysis.

Next, we again split up the Consistent and Inconsistent condition according to the presentation of the sentence sets (20 versus 40 seconds; **Table S1**). From the regular SPM analysis, the contrast of Consistent 20 seconds > Consistent 40 seconds revealed significant activation in the insula, while the contrast of Inconsistent 40 seconds > Inconsistent 20 seconds showed activations in the precentral gyrus and mPFC. For the first-level parametric analysis, we found that activations in the cerebellar Crus 2 (ROI analysis, MNI coordinate: 12 -76 -36, Pu et al., 2020), cerebellar lobule Ⅸ, caudate, inferior occipital gyrus and calcarine gyrus positively correlated with confidence ratings in the Inconsistent 20 seconds condition, while the caudate and lingual gyrus positively correlated with confidence ratings in the Inconsistent 40 seconds. No significant brain activations were found for second-level regression analyses on metacognitive sensitivity (e.g., meta-ratio). Taken together, as hypothesized, the results revealed some relationships with the mPFC and posterior cerebellum (Crus 2) in the Inconsistent condition, but the duration at which this happened (40 and 20 seconds respectively) differed.

**Discussion**

***Retrieval and metacognitive confidence***

We found no cerebellar activation during retrieval of sentence order. This replicates the previous study by Pu et al. (2020) which demonstrated that sequence retrieval did not activate the posterior cerebellum. These results are in line with the sequence detection hypothesis, which posits that the cerebellum is mainly involved in reducing prediction errors during sequence learning (Caligiore, Arbib, Miall, & Baldassarre, 2019). Therefore the cerebellum is expected to recruit the greatest activation during learning novel sequences, but not on retrieval when sequence learning is completed. Additionally, we found that cortical activation in the precuneus was correlated with retrieval accuracy in the Consistent 20 seconds condition; the precuneus is known to be involved in retrieving episodic memory (Lundstrom, Ingvar, & Petersson, 2005).

For metacognitive confidence on sequence retrieval, our results revealed brain activations of metacognitive tasks in the medial prefrontal (mPFC) for the contrast Inconsistent 40 > Inconsistent 20 seconds, and in the insula for the reverse contrast. This was consistent with previous studies of metacognition that have indicated a domain-general network involving the mPFC and insula (Morales et al., 2018; Vaccaro & Fleming, 2018). Parametric analyses of trial-by-trial confidence ratings further showed a positive correlation with the posterior cerebellar Crus 2 in the Inconsistent 20 seconds condition. These results are consistent with our hypothesis that metacognitive confidence on sequence retrieval recruits domain-general areas (e.g., mPFC) together with local areas (e.g., cerebellum) for specific tasks (McCurdy et al., 2013; Pu et al., 2020; Vaccaro & Fleming, 2018), such as retrieving action sequences in the current study. Although it is difficult to understand why there were different activations at different sentence durations, this pattern largely replicates the results by Pu et al. (2020). That study found an association between retrieval confidence and the posterior cerebellar Crus at a trial-by-trial level on when sentences were presented for 20 seconds, and at the individual level when sentences were presented for 40 seconds. Additionally, our findings revealed a positive correlation between activation in the striatum (e.g., caudate) and confidence ratings, which is compatible with previous studies that indicated domain-general signals in the striatum modulated by confidence level (Guggenmos, Wilbertz, Hebart, & Sterzer, 2016; Morales et al., 2018).

**Table S1:** Whole-brain analysis of sequence retrieval and confidence rating on retrieving sequences

| **Contrasts and Anatomical Label** | | ***MNI coordinate*** | | | ***Voxels*** | ***max t*** |
| --- | --- | --- | --- | --- | --- | --- |
|  |  | *x* | *y* | *z* |  |  |
|  | **Retrieval-related activity: first level whole-brain analyses** | | | | | |
| C | **Consistent 20 Seconds > Consistent 40 Seconds / Inconsistent 40 Seconds > Inconsistent 20 Seconds** | | | | | |
|  | **---** |  |  |  |  |  |
|  | **Consistent 40 Seconds > Consistent 20 Seconds** | | | | | |
|  | R Cerebellum (Ⅷ) | 8 | -68 | -42 | 119 | 4.27* |
|  | **Inconsistent 20 Seconds > Inconsistent 40 Seconds** | | | | | |
|  | L Postcentral Gyrus | -52 | -12 | 46 | 420 | 4.46*** |
|  | R Insula | 46 | 4 | -8 | 754 | 5.13*** |
|  | L Medial Temporal Gyrus | -48 | 10 | -32 | 129 | 4.91* |
|  | L Superior Frontal Gyrus | -12 | 44 | 46 | 124 | 4.43* |
|  | **Retrieval-related activity: first level parametric analyses by accuracy, positive correlation** | | | | | |
|  | **Consistent condition** | | | | | |
|  | Calcarine Gyrus | -6 | -84 | 2 | 524 | 5.00*** |
|  | **Inconsistent condition** | | | | | |
|  | --- |  |  |  |  |  |
|  | **Consistent 20 Seconds** | | | | | |
|  | R Precuneus | 12 | -58 | 36 | 271 | 4.28*** |
|  | **Consistent 40 Seconds/Inconsistent 40 Seconds** | | | | | |
|  | --- |  |  |  |  |  |
|  | **Inconsistent 20 Seconds** |  |  |  |  |  |
|  | L Cuneus | -16 | -68 | 22 | 170 | 4.94*** |
|  | L Posterior-Medial Frontal | 0 | -6 | 70 | 116 | 5.46* |
|  | **Retrieval-related activity: second level regression analyses by accuracy** | | | | | |
|  | **Consistent 20 Seconds / Consistent 40 Seconds / Inconsistent 20 Seconds** | | | | |  |
|  | **---** |  |  |  |  |  |
|  | **Inconsistent 20 Seconds** |  |  |  |  |  |
|  | R Superior Parietal Lobule | 18 | -48 | 58 | 268 | 5.45*** |
|  | **Confidence-related activity (retrieving sequences)：whole brain analysis** | | | | | |
|  | **Consistent 20 Seconds > Consistent 40 Seconds** | | | | | |
|  | R Insula | 40 | 12 | 2 | 170 | 4.23*** |
|  | **Consistent 40 Seconds > Consistent 20 Seconds /Inconsistent 20 Seconds > Inconsistent 40 Seconds** | | | | | |
|  | --- |  |  |  |  |  |
|  | **Inconsistent 40 Seconds > Inconsistent 20 Seconds** | | | | | |
|  | L Precentral Gyrus | -38 | -20 | 62 | 333 | 4.34*** |
|  | L Superior Medial Gyrus (mPFC) | -10 | 44 | 44 | 137 | 4.44* |
|  | **Confidence-related activity (retrieving sequences): first-level parametric analyses by confidence, positive correlation** | | | | | |
|  | **Consistent condition** | | | | | |
|  | Lingual Gyrus | 12 | -78 | -2 | 814 | 6.46*** |
|  | Precentral Gyrus | 38 | -16 | 50 | 152 | 5.88* |
|  | **Inconsistent condition** | | | | | |
|  | Lingual Gyrus | 14 | -80 | -12 | 3742 | 8.50*** |
|  | L Middle Occipital Gyrus | -36 | -74 | 20 | 164 | 5.05* |
|  | R Middle Temporal Gyrus | 48 | -72 | -2 | 159 | 4.82* |
|  | Caudate Nucleus | 18 | -20 | 22 | 192 | 4.88* |
|  | Precentral Gyrus | 46 | -14 | 60 | 174 | 6.52* |
|  | L Hippocampus | -36 | -14 | -16 | 187 | 5.97* |
|  | Caudate Nucleus | 12 | 18 | -4 | 1604 | 7.53*** |
|  | **Inconsistent > Consistent** |  |  |  |  |  |
|  | R Caudate | 14 | 16 | -4 | 262 | 5.30* |
|  | R Caudate | 12 | 22 | 4 |  | 3.44* |
|  | **Consistent > Inconsistent** |  |  |  |  |  |
|  | --- |  |  |  |  |  |
|  | **Consistent 20 Seconds** | | | | | |
|  | --- |  |  |  |  |  |
|  | **Consistent 40 Seconds** |  |  |  |  |  |
|  | Middle Occipital Gyrus | 28 | -90 | 18 | 351 | 6.41*** |
|  | **Inconsistent 20 Seconds** | | | | | |
|  | R Calcarine Gyrus | 12 | -80 | 2 | 5081 | 8.94*** |
|  | L Inferior Occipital Gyrus | -42 | -74 | -10 | 203 | 4.99*** |
|  | R Cerebellum (Ⅸ) | 14 | -44 | -44 | 137 | 5.41* |
|  | R Caudate | 10 | 16 | 0 | 461 | 6.48*** |
|  | **ROI**: R Cerebellum (Crus 2) | 8 | -76 | -36 | 222 | 5.64** |
|  | **Inconsistent 40 Seconds** |  |  |  |  |  |
|  | R Lingual Gyrus | 16 | -80 | -2 | 605 | 5.58*** |
|  | R Caudate | 14 | 14 | -6 | 712 | 7.10*** |
|  | **Confidence-related activity (retrieving sequences): second-level regression analyses by meta-ratio** | | | | | |
|  | --- |  |  |  |  |  |

**Notes:** Coordinates refer to the MNI (Montreal Neurological Institute) stereotaxic space. Whole-brain analysis thresholded at voxel-wise uncorrected p < 0.001 with cluster-wise FWE corrected p < 0.05, with voxel extent ≥ 10. Only the highest peaks of each cluster are shown. L = left, R = right.

* *p* < 0.05, ***p* < 0.01, ****p* < 0.001 (cluster-level FWE corrected; for ROI: cluster-level FWE corrected using a small volume correction with a sphere with 15 mm radius and centered around a priori MNI coordinate [12 -76 -36]).

**References**

Bègue, I., Vaessen, M., Hofmeister, J., Pereira, M., Schwartz, S., & Vuilleumier, P. (2019). Confidence of emotion expression recognition recruits brain regions outside the face perception network. *Social Cognitive and Affective Neuroscience*, *14*(1), 81–95. https://doi.org/10.1093/scan/nsy102

Caligiore, D., Arbib, M. A., Miall, R. C., & Baldassarre, G. (2019). The super-learning hypothesis: Integrating learning processes across cortex, cerebellum and basal ganglia. *Neuroscience and Biobehavioral Reviews*, *100*, 19–34. https://doi.org/10.1016/j.neubiorev.2019.02.008

Fleming, S. M., & Lau, H. C. (2014). How to measure metacognition. *Frontiers in Human Neuroscience*, *8*(July), 1–9. https://doi.org/10.3389/fnhum.2014.00443

Guggenmos, M., Wilbertz, G., Hebart, M. N., & Sterzer, P. (2016). Mesolimbic confidence signals guide perceptual learning in the absence of external feedback. *eLife*, *5*(MARCH2016), 1–19. https://doi.org/10.7554/eLife.13388

Lundstrom, B. N., Ingvar, M., & Petersson, K. M. (2005). The role of precuneus and left inferior frontal cortex during source memory episodic retrieval. *NeuroImage*, *27*(4), 824–834. https://doi.org/10.1016/j.neuroimage.2005.05.008

Maniscalco, B., & Lau, H. (2012). A signal detection theoretic approach for estimating metacognitive sensitivity from confidence ratings. *Consciousness and Cognition*, *21*(1), 422–430. https://doi.org/10.1016/j.concog.2011.09.021

McCurdy, L. Y., Maniscalco, B., Metcalfe, J., Liu, K. Y., de Lange, F. P., & Lau, H. (2013). Anatomical Coupling between Distinct Metacognitive Systems for Memory and Visual Perception. *Journal of Neuroscience*, *33*(5), 1897–1906. https://doi.org/10.1523/JNEUROSCI.1890-12.2013

Morales, J., Lau, H., & Fleming, S. M. (2018). Domain-General and Domain-Specific Patterns of Activity Supporting Metacognition in Human Prefrontal Cortex. *The Journal of Neuroscience*, *38*(14), 3534–3546. https://doi.org/10.1523/jneurosci.2360-17.2018

Pu, M., Heleven, E., Delplanque, J., Gibert, N., Ma, Q., Funghi, G., & Van Overwalle, F. (2020). The posterior cerebellum supports the explicit sequence learning linked to trait attribution. *Cognitive, Affective and Behavioral Neuroscience*, *20*(4), 798–815. https://doi.org/10.3758/s13415-020-00803-7

Vaccaro, A. G., & Fleming, S. M. (2018). Thinking about thinking: A coordinate-based meta-analysis of neuroimaging studies of metacognitive judgements. *Brain and Neuroscience Advances*, *2*, 239821281881059. https://doi.org/10.1177/2398212818810591
